# Computational Studies to Understand the Neuroprotective Mechanism of Action Basil Compounds

**DOI:** 10.3390/molecules28207005

**Published:** 2023-10-10

**Authors:** Varinder Singh, Somdutt Mujwar, Manjinder Singh, Tanveer Singh, Sheikh F. Ahmad

**Affiliations:** 1 Department of Pharmaceutical Sciences and Technology, Maharaja Ranjit Singh Punjab Technical University, Bathinda 151001, Punjab, India; 2 Chitkara College of Pharmacy, Chitkara University, Rajpura 140401, Punjab, India; 3 Department of Neuroscience and Experimental Therapeutics, College of Medicine, Texas A&M Health Science Center, College Station, TX 77807, USA; tanveersingh1988@gmail.com; 4 Department of Pharmacology and Toxicology, College of Pharmacy, King Saud University, Riyadh 11451, Saudi Arabia

**Keywords:** basil compounds, neuroprotection, molecular docking, molecular dynamics, density functional theory (DFT), neurodegenerative diseases

## Abstract

Neurodegenerative diseases, such as Alzheimer’s and Parkinson’s, pose a significant global health challenge, emphasizing the need for novel neuroprotective agents. Basil (*Ocimum* spp.) has been recognized for its therapeutic potential, and numerous studies have reported neuroprotective effects. In this manuscript, we present a computational protocol to extricate the underlying mechanism of action of basil compounds in neuroprotective effects. Molecular docking-based investigation of the chemical interactions between selected bioactive compounds from basil and key neuroprotective targets, including AChE, GSK3β, γ-secretase, and sirtuin2. Our results demonstrate that basil compound myricerone caffeoyl ester possesses a high affinity of −10.01 and −8.85 kcal/mol against GSK3β and γ-secretase, respectively, indicating their potential in modulating various neurobiological processes. Additionally, molecular dynamics simulations were performed to explore the protein–ligand complexes’ stability and to analyze the bound basil compounds’ dynamic behavior. This comprehensive computational investigation enlightens the putative mechanistic basis for the neuroprotective effects of basil compounds, providing a rationale for their therapeutic use in neurodegenerative disorders after further experimental validation.

## 1. Introduction

Dementia is a progressive neurodegenerative disorder that mainly affects the consciousness content instead of the consciousness level. The most common form of dementia is refractory dementia, which comprises Alzheimer’s disease (AD) and vascular dementia (VD). Pathologically, dementia is a multi-etiological disorder in which many interrelated biochemical changes happen, causing symptoms like poor memory and forgetfulness. Among various identified pathological factors, abnormal depositions of amyloid beta (Aβ) plaques and tau proteins in the brain have been known to be primarily responsible for dementia and related neurodegeneration. Apart from this, there are many added factors to the origin of dementia, such as excess glycemic load, hampered cholinergic neurotransmission due to elevated acetylcholinesterase activity, oxidative stress, and inflammation [1,2]. Certain evidence proves the effect of oxidative impairment for the endorsement of amyloid aggregates and NFT formation in Alzheimer’s-type dementia [3]. The large amount of Fe^2+^ and Cu^2+^ in the brain hasten the ROS formation, which further causes Aβ neurotoxicity. Also, the high receptive carbonyls and oxygen radicals levels lead to AGEs, which cross-linked and caused glycation of the tau and Aβ or proteins, ultimately inducing the neuron’s cell death [4]. Various macromolecular targets like AChE, GSK3β, γ-secretase, and sirtuin2 are found to have a major influential role in managing neuronal signaling, which is directly correlated with the pathogenesis of neurodegeneration [5,6].

It has been observed that most of the research is focused on developing those molecules that could target one aspect of dementia [7,8]. Such single target-oriented drugs only enable a palliative treatment rather than curing or preventing neurodegenerative multifactorial AD. This could be one of the reasons for the limited success of synthetic analogs in clinical practice. Thus, a promising strategy to manage dementia could involve the implementation of a multitarget/multidrug protocol. Due to their diverse phytochemical profiles, medicinal herbs have been traditionally employed as native multidrug formulations in various therapeutic practices, exhibiting the advantage of minimal to no observed side effects [9,10,11].

*Ocimum basilicum* L., commonly known as basil, is a popular culinary herb with a rich history of traditional medicinal use. Belonging to the Lamiaceae family, it is native to regions in Asia and Africa. Basil is characterized by its vibrant green leaves, distinctive aroma, and pleasant flavor, making it a widely cherished ingredient in various cuisines worldwide [12,13,14,15]. Aside from its culinary applications, *O. basilicum* possesses several potential health benefits due to its phytochemical composition. It contains essential oils, flavonoids, and phenolic compounds, contributing to its antioxidant and anti-inflammatory properties [16,17,18,19,20]. These bioactive compounds have been associated with potential neuroprotective, hepatoprotective, antimicrobial, and immunomodulatory effects. Moreover, traditional medicine has used basil to alleviate certain ailments such as brain disorders, digestive issues, respiratory problems, and stress-related conditions. Recently, we reported two potent flavones from basil, which showed the ability to modulate various crucial pathological targets of dementia, such as the cholinergic system, oxidative stress, and inflammation, revealing their multi-targeted approach to treating dementia. Studies have shown the presence of more than 80 compounds related to different classes of phytoconstituents in basil [21,22,23,24,25]. Thus, it is hypothesized that basil could have potential neuroprotective compounds. However, investigations studying the detailed effect of basil components on the crucial biochemical targets such as AChE, GSK3β, γ-secretase, and sirtuin2 of dementia are scanty [26,27,28,29,30,31,32,33]. Thus, the present study was designed to study the detailed mechanism of action of previously identified bioactive compounds in basil using silico techniques.

## 2. Results and Discussion

### 2.1. Design of Ligand Library

Ligands having diverse chemical functions like phenolic acid, glycosides, sterols, triterpenes, kavalactones, flavonoids, catechin, etc., were identified from basil that were traditionally used for the management and treatment of cancer and related conditions. The detailed information related to the source and part of the plant for the specific chemical constituent considered in the current study were tabulated in Table S1, presented in the Supplementary Material. These chemical constituents generated an herbal-based ligand library of 122 compounds. Isomeric SMILES for all the ligands were obtained from the PubChem database to develop their two-dimensional structure using ChemDraw9.0 [34]. These prepared ligand structures undergo the energy minimization process for developing their three-dimensional structure required for executing virtual screening against the macromolecular targets.

### 2.2. Target Selection

AChE, an enzyme responsible for regulating the degradation of the ACh neurotransmitter into acetate and choline, can result in neurodegenerative symptoms when it is overexpressed. The 3D structural model of the AChE enzyme complex with inhibitor donepezil was attained from PDB (PDB code: 4ey7) [35,36]. The X-ray diffraction (XRD) method resolved the crystallized macromolecular complex assembly with a 2.35 Å resolution. The macromolecular receptor has a dimeric chain of 542 amino acids, out of which one chain was deleted to get a monomeric receptor. The bound ligands were deleted from the monomeric chain to obtain the nascent receptor required for the docking study. γ-Secretase is an enzyme that plays a pivotal role in governing the cleavage of cellular proteins, including APP, which is essential for the production of amyloid tau proteins. These proteins have been implicated in the neurodegenerative processes observed in Alzheimer’s disease and related dementia disorders. The three-dimensional structural model of γ-secretase enzyme complex with an inhibitor DAPT was attained from PDB (PDB code: 5fn2). The electron microscopy (EM) method resolved the crystallized macromolecular complex assembly with a 4.20 Å resolution. The macromolecular receptor has a monomeric subunit of γ-secretase, having 265 amino acids. The bound ligand DAPT was deleted from the monomeric chain to obtain the nascent receptor required for the docking study. GSK3β activates the nuclear factor-kappa B (NF-κB) required to produce pro-inflammatory cytokines that can exacerbate neurodegeneration. The three-dimensional structural model of the GSK3β enzyme complex with an inhibitor ARN25068 was attained from PDB (PDB code: 7oy5). The XRD method resolved the crystallized assembly of the macromolecular complex with a 2.57 Å resolution. The macromolecular receptor has a dimeric chain of 351 amino acids, out of which one chain was deleted to obtain a monomeric receptor. The bound ligands were deleted from the monomeric chain to obtain the nascent receptor required for the docking study. SIRT2 has been implicated in the pathogenesis of several neurodegenerative diseases and can be inhibited to control the process of neurodegenerative pathogenesis. The three-dimensional structural model of the SIRT2 enzyme complex with a selective inhibitor (8NO) was attained from PDB (PDB code: 5y5n). The XRD method resolved the crystallized assembly of the macromolecular complex with a 2.30 Å resolution. The macromolecular receptor has a dimeric chain of 336 amino acids, out of which one chain was deleted to obtain a monomeric receptor. The bound ligand was deleted from the monomeric chain to obtain the nascent receptor required for the docking study.

### 2.3. Molecular Docking Studies

**Figure 1:**
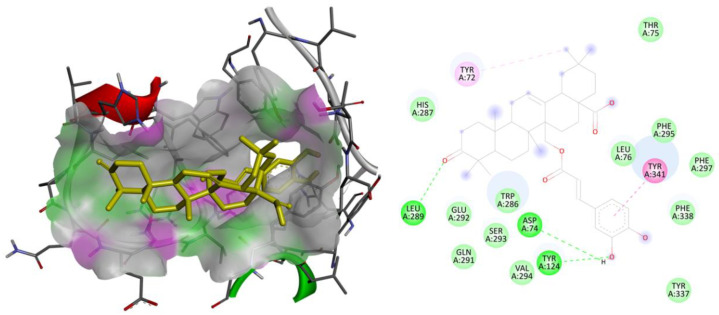
Binding interactions: two and three-dimensional interactions of the ligand MCE with the human AChE receptor.

**Figure 2:**
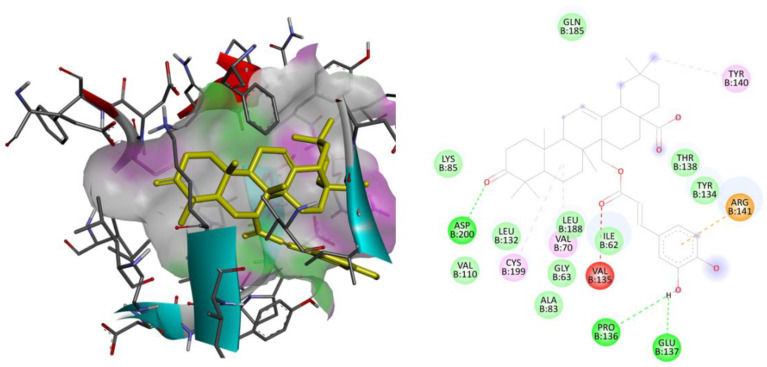
Binding interactions: two and three-dimensional interactions of the ligand MCE with the human GSK3β receptor.

The three-dimensional models of AChE, gamma-secretase, GSK3β, and the SIRT2 receptor were subjected to redocking with their respective reference ligands, aiming to confirm the reliability of the applied docking methodology. Validation of the utilized docking protocols was completed for all the considered therapeutic targets as the observed binding free energy of docked reference ligands was well within the range of −5 to −15 kcal/mol with identical interactions as those were observed in the bioactive crystallized conformations. The docked conformations for each reference ligand are flawlessly overlaying over their bioactive crystallized conformations. These considered parameters for the validation purpose indicate that the utilized docking parameters are effective and that the docking software is exactly simulating the ligand–receptor complexation process that is taking place within the human body. The ligand library that was created underwent additional screening against therapeutic targets associated with human cancer-related physiological processes. Following the outcome of the virtual screening, the best lead molecule was picked based on the lowest binding energy and the chemical interactions observed between the ligand and the macromolecular target. The binding energy for each ligand against the human AChE, gamma-secretase, GSK3β, and SIRT2 receptor was tabulated in Table 1. The 2D and 3D binding interactions of the ligand MCE with the human AChE receptor and with human GSK3β receptor given in Figure 1 and Figure 2, respectively.

### 2.4. Molecular Dynamic Simulation

**Figure 3:**
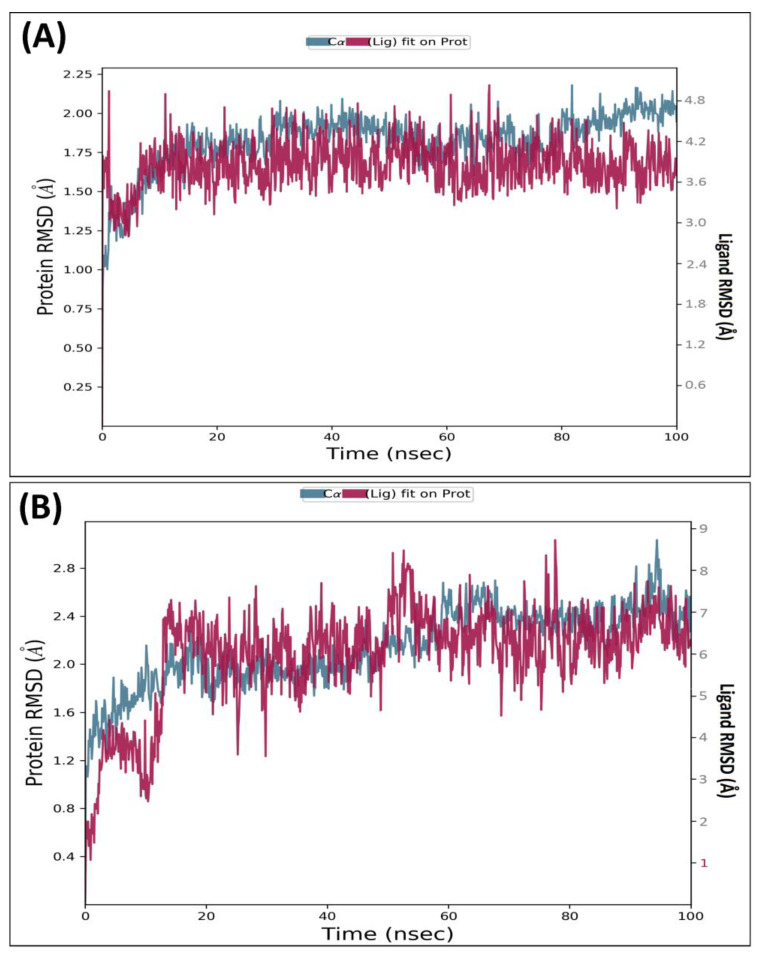
Root Mean Square Deviation: observed RMSD for Cα chain of AChE complexed with ligand MCE (**A**) and GSK3β complexed with ligand MCE (**B**) detected while executing 100 ns MD simulation.

The docking analysis of the herbal-based ligand library against neurodegenerative macromolecular targets involved in diseases has revealed that the therapeutic potential of the sweet basil plant is because of MCE. MCE is exerting its neuroprotective effect via interacting with three of four targets in the ongoing research. Thus, the macromolecular complex of MCE with human AChE, gamma-secretase, and GSK3β receptor were shortlisted to execute MD simulation for 100 ns to evaluate their thermodynamic stability with time. The MD simulation analysis revealed that the ligand MCE was highly stabilized within the macromolecular cavity of AChE and GSK3β throughout the simulation time. At the same time, it shows some fluctuations within the cavity of SIRT2. The drug–receptor complex should be stable enough, at least for the nano-second time range, for the execution of the therapeutic response. MD analysis has revealed that the target AChE has a monomeric chain of 530 amino acids. AchE receptor constitutes 4130 heavy atoms out of a total of 8148 atoms. The complex MCE ligand has 9 rotatable bonds with 46 heavy atoms out of a total of 98 atoms in total. RMSD analysis during the 100 ns timeframe was executed to reveal the thermodynamic stability and structural variations of the macromolecular backbone. The trajectories obtained for the human AChE receptor complexed with ligand MCE showed that the complex was highly stable throughout the 100 ns simulation. Both the C_α_ protein backbone and the complex MCE showed high stability. RMSD value observed for the macromolecular backbone ranging from 1.25 to 2.25 Å, while the complex ligand MCE showed the RMSD value in a 3.6–4.2 Å within the receptor’s cavity. MD simulation of 100 ns for another complex of human GSK3β receptor with MCE was stable throughout the simulation. The GSK3β has a monomeric chain of 344 amino acids. GSK3β receptor constitutes 2626 heavy atoms out of a total of 5273 atoms. The trajectories obtained for the human GSK3β receptor complexed with MCE showed that the complex was highly stable throughout the 100 ns simulation. The C_α_ protein chain and the complex compound–36 showed very little fluctuation to achieve the stabilized conformation. RMSD value observed for the protein backbone remains stabilized throughout, ranging from 1.5 to 2.5 Å. In comparison, the complex MCE showed a couple of initial fluctuations to achieve the stabilized conformation with the RMSD value in a range of 6.0–8.0 Å within the receptor’s cavity. Figure 3 illustrates the observed RMSD of the human AChE macromolecular chain and the complex compound MCE (A) and GSK3βcomplexed with compound MCE (B), respectively.

**Figure 4:**
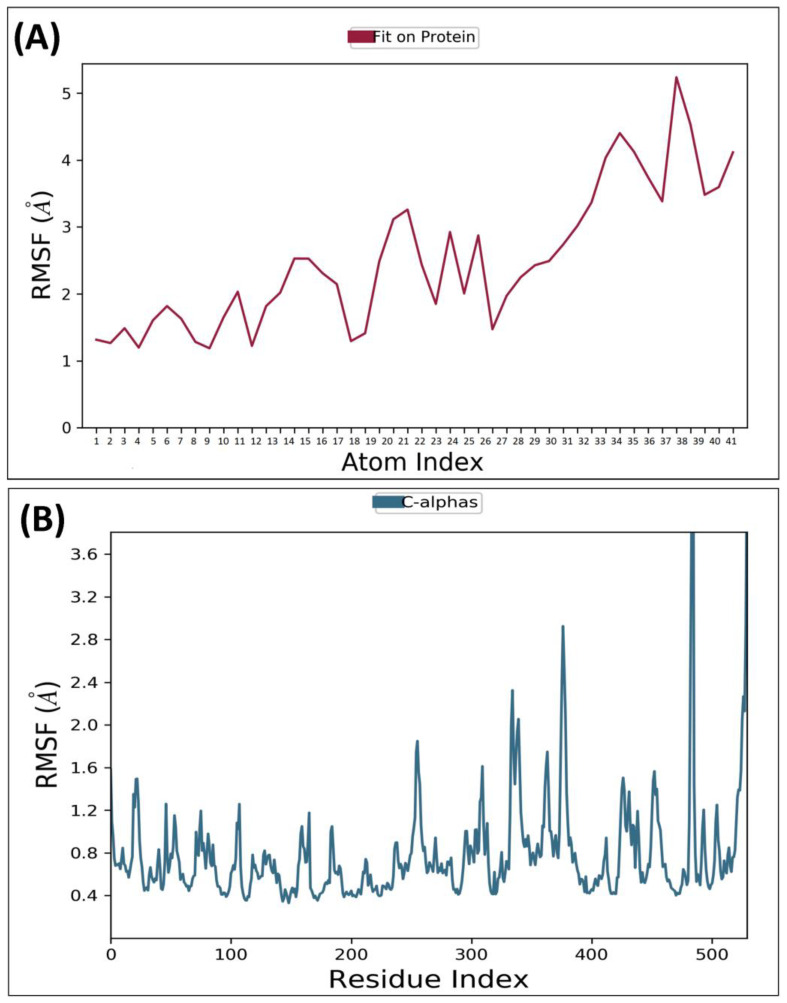
Root Mean Square Fluctuation: observed RMSF for AChE complexed with compound MCE detected while executing 100 ns MD simulation.

**Figure 5:**
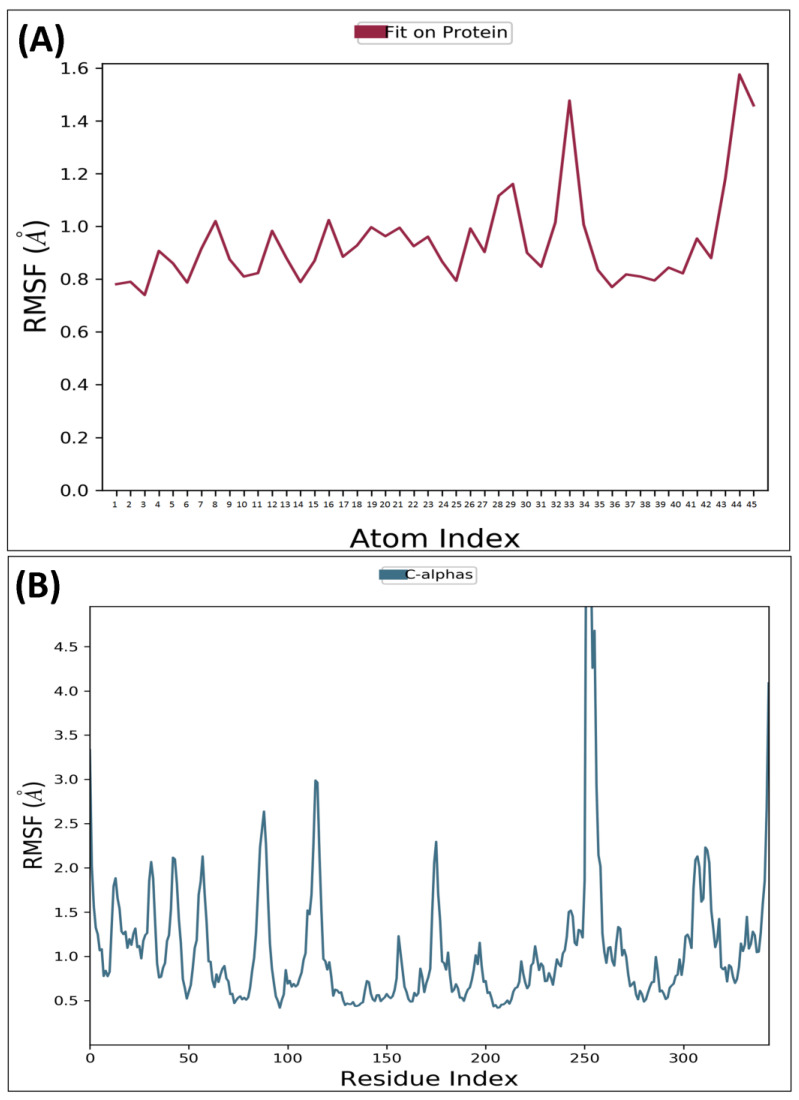
Root Mean Square Fluctuation: observed RMSF for GSK3β complexed with compound MCE detected while executing 100 ns MD simulation.

RMSF is a metric that measures the deviation of the atoms in a protein or ligand structure from their average position. It is a valuable tool in assessing the dynamics and flexibility of a protein or ligand molecule. The significance of protein RMSF lies in its ability to provide information about the relative flexibility of different parts, which can be useful in predicting protein dynamics and assessing stability. MD analysis for AChE receptor complexed with ligand MCE revealed that the protein backbone’s RMSF was within 0.4–1.4Å odds except for some terminal residues, while for MCE, it ranged from 0.5–1.5Å. RMSF for GSK3β receptor complexed with compound MCE was revealed to be within 0.5–2.0 Å, while the compound–36 average variation was found to be within 1.5–4.5Å, confirming the stability of both macromolecule and the complex ligand within the macromolecular cavity. RMSF of the AChE backbone and complexed compound MCE observed during MD analysis was depicted in Figure 4A,B, while the GSK3β receptor complexed with compound MCE was depicted in Figure 5A,B.

**Figure 6:**
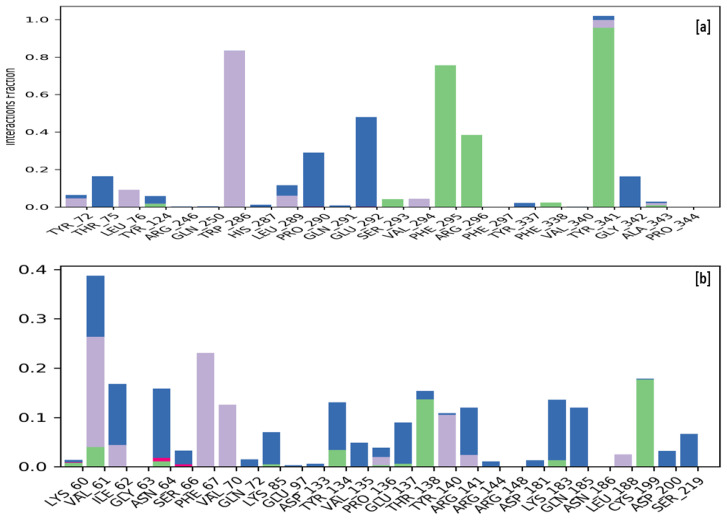
Protein–ligand contacts: protein–ligand interactions identified between the ligand MCE with human AChE receptor (**a**) and GSK3β receptor (**b**). The interactions were visualized using different colored bars, with green representing hydrogen bonds, blue representing water bridges, and purple representing hydrophobic interactions.

The stability of a protein–ligand complex is attributed to the formation of hydrogen bonds, hydrophobic contacts, and ionic interactions during an MD simulation. The strength of these interactions was continuously monitored throughout the simulation to evaluate the ligands’ stability in both the macromolecular complexes. During the simulation procedure, the interaction between the compound MCE and the human AChE receptor shows hydrophobic interactions with the amino acids Tyr72, Leu76, Trp286, Leu289, and Val294, whereas Ser293, Phe295, Arg296, Phe338, and Tyr341 are forming a hydrogen bond while residues Thr75, Pro290, Glu292, and Gly342 were interacting via water bridges with the compound MCE. The simulation of GSK3β complexed with compound MCE has revealed that the ligand is interacting with the macromolecular receptor via hydrophobic interactions with the amino acids Val61, Phe67, Val70, Pro136, Tyr140, and Leu188, whereas Thr138 and Cys199 are involved via a hydrogen bond. In contrast, residues Ile62, Asn64, Ser66, Gln72, Lys85, Tyr134, Val135, Glu137, Arg141, Arg144, Asp181, Cys183, Gln185, Asp201, and Ser219 were involved via formation of a water bridge with the compound MCE. Figure 6 illustrates the interactions of ligand MCE with the active residues of the human AChE receptor (a) and the GSK3β receptor (b).

## 3. Materials and Methodology

### 3.1. Ligand Library Preparation

A library of 40 ligands from the major chemical constituents of the sweet basil plant belonging to the *Ocimum* family was created. Based upon research from a wide range of literature, it was revealed that the sweet basil plant has been used for treating and managing neurodegenerative disorders [37,38,39]. The concerned plants have a long history of being used to treat and manage neurodegeneration and related disorders since the traditional times. It is hoped that this diverse array of ligands from the sweet basil plant will lead to the identification of a highly effective agent in the management of neurodegenerative disorders like AD, as well as a better understanding of the physiological mechanisms involved in their therapeutic effect for the same [40,41,42,43].

### 3.2. Macromolecular Target Selection and Preparation

Macromolecular targets like AChE, GSK3β, γ-secretase, and sirtuin2 are found to have a major influential role in the management of the concentration of neurotransmitters which is directly correlated with the pathogenesis of neurodegeneration [5,6]. AChE plays a crucial role in regulating cholinergic neurotransmission, and its dysregulation can contribute to the development of neurodegenerative diseases. Acetylcholinesterase (AChE) is an enzyme that plays a crucial role in the nervous system’s degradation of the neurotransmitter acetylcholine (ACh). In diseases such as Alzheimer’s disease, Parkinson’s disease, and amyotrophic lateral sclerosis, the activity of AChE is significantly upregulated [44,45]. GSK3β is a serine/threonine kinase involved in numerous cellular processes, including glycogen metabolism, cell cycle regulation, and neuronal development. Studies have shown that GSK3β activity is increased in Alzheimer’s disease, Huntington’s disease, and Parkinson’s. In Alzheimer’s disease, GSK3β promotes the hyperphosphorylation of tau protein, leading to neurofibrillary tangles, a disease hallmark. Additionally, GSK3β can promote the accumulation of beta-amyloid peptides, leading to the formation of amyloid plaques in Alzheimer’s disease. In Parkinson’s disease, GSK3β activity contributes to the degeneration of dopaminergic neurons in the substantia nigra. GSK3β can promote alpha-synuclein aggregation, leading to Lewy bodies forming, characteristic of Parkinson’s disease [46,47]. Furthermore, GSK3β can activate the pro-inflammatory transcription factor nuclear factor-kappa B (NF-κB), producing pro-inflammatory cytokines that can exacerbate neurodegeneration. In conclusion, GSK3β plays a crucial role in the pathogenesis of neurodegenerative diseases, and its inhibition can infer the potential therapeutic benefits in treating neurodegenerative diseases. Gamma-secretase is a transmembrane protease complex that cleaves various membrane proteins, including amyloid precursor protein (APP), Notch, and ErbB4. The cleavage of APP by gamma-secretase generates beta-amyloid peptides, which are the main constituents of amyloid plaques in Alzheimer’s disease [48,49,50]. Thus, gamma-secretase plays a crucial role in the pathogenesis of neurodegenerative diseases by generating beta-amyloid peptides and cleaving other crucial proteins involved in neuronal signaling. Sirtuin–2 (SIRT2) is a member of the sirtuin family of NAD(+)-dependent deacetylases that play important roles in cellular metabolism and stress response. SIRT2 is highly expressed in the brain and has been implicated in the pathogenesis of several neurodegenerative diseases, including Parkinson’s disease, Alzheimer’s disease, and Huntington’s disease. SIRT2 plays a critical role in the pathogenesis of neurodegenerative diseases by promoting the accumulation of toxic protein aggregates and dysregulating the immune response in the brain [51,52]. Thus, SIRT2 has been proposed as a potential therapeutic target for treating neurodegenerative diseases.

A three-dimensional structure model of a human AChE receptor complexed with inhibitor donepezil was downloaded from the protein databank (pdb id: 4ey7) [35,36]. The downloaded AChE and complexed inhibitor donepezil were separated by deleting both of them one by one to generate nascent receptor and ligand molecules to execute docking analysis. Three-dimensional structure model of human γ-secretase complexed with dipeptides inhibitor N-[N-(3,5-difluorophenacetyl)-L-alanyl]-S-phenyl glycine t-butyl ester (DAPT) was downloaded from protein databank (pdb id: 5fn2) [53,54]. The downloaded γ-secretase and complexed inhibitor DAPT were separated by deleting them one by one to generate nascent receptor and ligand molecules to execute docking analysis. A three-dimensional structure model of the human GSK3β receptor complexed with inhibitor ARN25068 was downloaded from the protein databank (pdb id: 7oy5) [55,56]. The downloaded GSK3β and complexed inhibitor ARN25068 were separated by deleting both of them one by one to generate nascent receptor and ligand molecules to execute docking analysis. A three-dimensional structure model of a human SIRT2 receptor complexed with a selective inhibitor was downloaded from a protein databank (pdb id: 5y5n) [57,58]. The downloaded SIRT2 and complexed inhibitor were separated by deleting both of them one by one to generate nascent receptor and ligand molecules to execute docking analysis.

### 3.3. Molecular Docking Studies

All the macromolecular targets in the current computational paradigm were re-docked with the separated reference ligand using Autodock software to validate the utilized docking protocol for each. Autodock Tools 4.2 uses the Lamarckian Genetic algorithm to dock ligands with macromolecular targets [59]. The validity of the docking parameters was tested by comparing the conformation and chemical similarity of the reference ligands with that of the active binding site of their respective macromolecular targets [59]. After verifying the parameters, they were used to computationally screen the library of prepared ligands against each of them with the intent to identify the most potent ligand responsible for the therapeutic effect against the neurodegenerative effect as well as to identify the most probable mechanism of action involved in the same [60,61].

### 3.4. Molecular Dynamics Simulation

Based upon the observed docking results and pharmacokinetic profiling, the macromolecular complex of Myricerone caffeoyl ester (MCE) ligand with AChE, gamma-secretase, and GSKβ3 receptor was shortlisted for executing MD simulations. Next, 100 ns MD simulation was performed for all the ligand MCE macromolecular complexes using the Desmond Schrodinger software module [62]. Desmond software by Schrodinger uses OPLS-2005 for executing MD simulation for drug-receptor complex. An atomic model of these complexes was prepared by adding explicit solvent molecules, neutralizing the system, and adding any necessary ions to reach neutrality [63,64]. The system’s energy minimization was done using the steepest descent algorithm to relax the system and remove any bad contacts or steric clashes between atoms. The system’s equilibrium was performed using a series of short, low-temperature constant pressure (NPT) simulations. The temperature gradually increases, and the system is subjected to positional restraints. This helps to ensure that the system is in a stable and equilibrated state before the actual simulation. The simulation is run for 100 ns to obtain the desired results by considering the coordinates of the atoms, the RMSD values, and the system’s energies. This helps to understand the dynamics and behavior of the system and gain insights into the structure and functional stability of the complex with time.

## 4. Conclusions

The increasing prevalence of neurodegenerative diseases such as Alzheimer’s and Parkinson’s underscores the urgent need for novel neuroprotective agents. Basil (*Ocimum* spp.) has emerged as a promising candidate due to its reported therapeutic potential and neuroprotective effects. In this manuscript, we have implemented a computational protocol to identify the most potent compound and elucidate the underlying mechanism of action of basil compounds in exhibiting neuroprotective effects. Docking-based investigations have revealed the MCE as a most potent basil compound based upon observed chemical interactions with key neuroprotective targets, including AChE, GSK3β, γ-secretase, and sirtuin2, possessing the potential to modulate crucial neurobiological processes in dementia and related disorders. Further, by employing molecular dynamics simulations, the stability of the protein–ligand complexes was confirmed by analyzing the dynamic behavior of the bound MCE with the multiple therapeutic targets, including AChE and GSK3β. These simulations provided valuable insights into the interactions between the basil compounds and their respective targets, further supporting their neuroprotective properties. The comprehensive computational investigation presented in this study sheds light on the putative mechanistic basis underlying the neuroprotective effects of basil compounds. The observed high affinity for key neuroprotective targets highlights their potential as promising candidates for therapeutic interventions in neurodegenerative disorders. However, further experimental validation is essential to solidify these findings and establish the translational potential of basil compounds for neuroprotection to offer a novel avenue for developing effective plant-based neuroprotective therapies in the battle against devastating neurodegenerative diseases.

## Figures and Tables

**Table 1:** Binding energy attained after virtual screening against the human AChE, gamma-secretase, GSK3β, and SIRT2 receptor.

Ligand	AChE (4ey7)	Gamma-Secretase (5fn2)	GSK3B (7oy5)	Sirtuin–2 (5y5n)
18HODA	−5.09	−2.28	−4.37	−7.06
2	−6.17	−2.74	−4.30	−7.56
Apigenin	−8.15	−5.72	−7.58	−9.33
Caffeic Acid	−4.63	−4.04	−5.18	−5.91
Caffosyl Gluco	−6.61	−3.64	−5.30	−8.05
Caftaric Acid	−4.52	−2.85	−3.65	−4.63
Chichoric Acid	−5.22	−2.43	−5.30	−6.66
DHDM Flavone	−8.52	−5.48	−7.45	−10.17
DH Palmitic Acid	−4.04	−1.91	−3.33	−5.95
E Arabinoside	−8.64	−5.36	−7.43	−7.61
Ellagic Acid Pentoside	−9.31	−5.78	−7.70	−8.47
Feruloyl TA	−4.27	−1.95	−4.20	−5.45
Ferusoyl Glucosidase	−6.39	−3.40	−4.45	−7.53
GQ Apioside	−6.97	−2.70	−4.97	+35.96
HOD Dienoic Acid	−5.32	−2.58	−4.16	−8.49
Isomeli A	−5.93	−3.27	−4.85	−8.78
Isoquercetin	−8.71	−4.80	−6.93	−7.77
KoGluco	−6.82	−3.30	−5.32	+1.83
Linolenic Acid	−5.47	−3.04	−4.00	−8.26
Lithospermica A	−5.32	−4.38	−6.77	−6.47
Myricerone caffeoyl ester	**−7.54**	**−8.85**	**−10.01**	**+17.06**
Octa DA	−5.41	−2.49	−4.10	−7.21
Octadeca TA	−6.13	−2.71	−4.96	−8.52
Olenoliec Acid	**−7.79**	**−7.96**	**−9.40**	**+10.64**
Palmitic Acid	−5.33	−2.30	−3.81	−6.72
Quercetin 3oA	−6.10	−3.72	−4.86	+0.23
QuercetinDG	−6.40	−3.21	−5.02	−1.42
Rosmar Acid 3 Gluco	−6.42	−1.70	−3.56	−4.42
Rosmarinic Acid	−6.27	−4.69	−6.29	−8.63
Rutin	−5.99	−4.22	−4.72	−3.08
Salicylic Acid Gluco	−4.53	−3.22	−3.96	−6.42
Salicylic AoG	−5.70	−3.57	−5.01	−6.43
Salvianolic Acid	−8.75	−5.68	−7.06	−11.50
Savialinic Acid	−8.78	−3.93	−5.87	−10.61
Saviolonic AA	−6.58	−4.29	−6.68	−9.25
Savialonic AC	−8.27	−6.00	−6.80	−10.75
THOctadecadienoic Acid	−4.22	−1.99	−3.55	−6.91
Vicenin2	−8.23	−3.48	−5.33	+39.13

Values in bold represent the highest active compounds with best Binding energy.

## Data Availability

The data presented in this study are available within the article.

## Supplementary Materials

The following supporting information can be downloaded at: https://www.mdpi.com/article/10.3390/molecules28207005/s1.
