# Supplementary material for: Computational Studies to Understand the Neuroprotective Mechanism of Action Basil Compounds
Source: Molecules. 2023 Oct 10;28(20):7005. doi: 10.3390/molecules28207005 (PMC10609097; doi:10.3390/molecules28207005)
Supplement: Supplementary file 1 [file molecules-28-07005-s001.zip › molecules-2594033-supplementary.pdf]

Table S1. Chemical constituents from the Basil plant.

Sr. No	Phytochemicals	Chemical Structures
1	4-Hydroxybenzoic acid	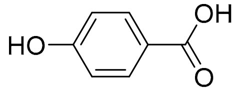
2	18-Hydroxyoctadecadienoic acid	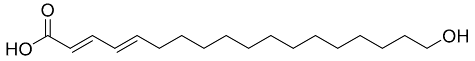
3	Apigenin-7-glycoside	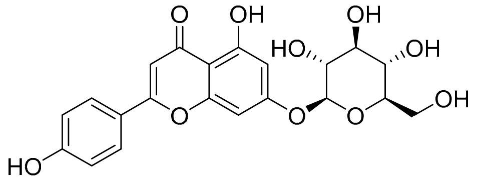
4	Caffeic acid	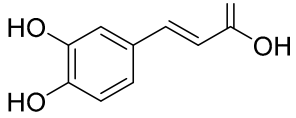
5	Caffosylglucoside	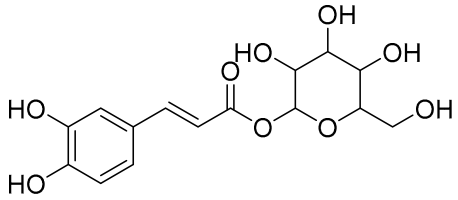
6	Caftaric acid	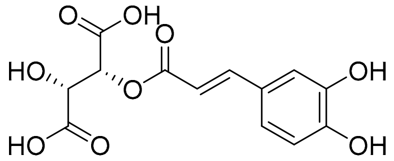

7	Chichoric acid	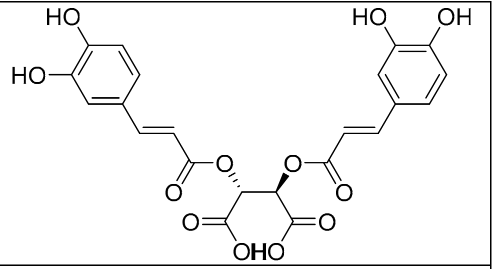
8	Dihydroxydimethoxy flavone	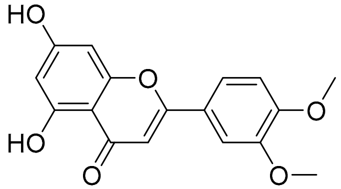
9	Dihydroxypalmitic acid	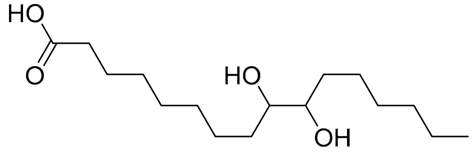
10	Ellagic acid arabinoside	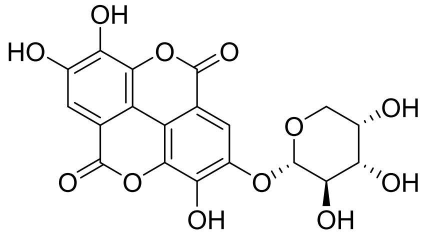

11	Ellagic acid pentoside	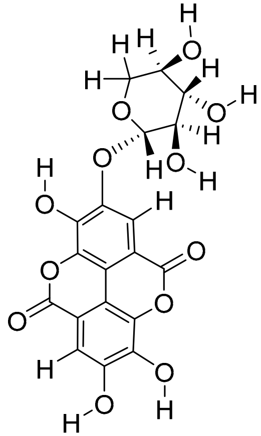
12	Feruloyltartaric acid	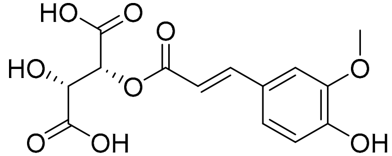
13	Glucoliquiritin apioside	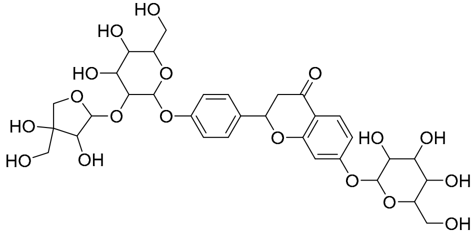
14	Hydroxydecadienoic acid	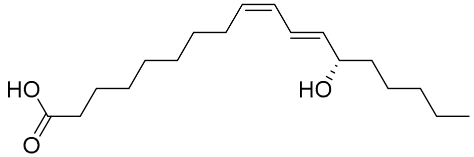
15	Isomelitrica acid	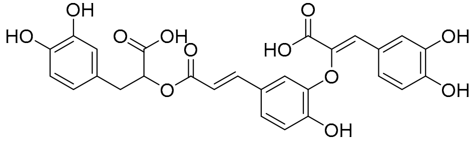

16	Isoquercetin	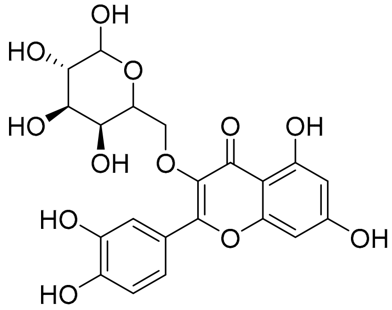
17	Kaempferol-o-glucoside	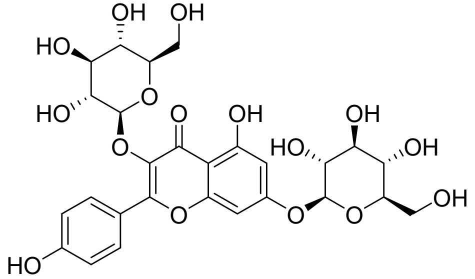
18	Linolenic acid	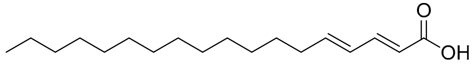
19	Lithospermic acid	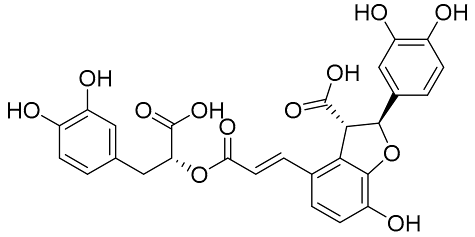

20	Myricerion caffoyl ester	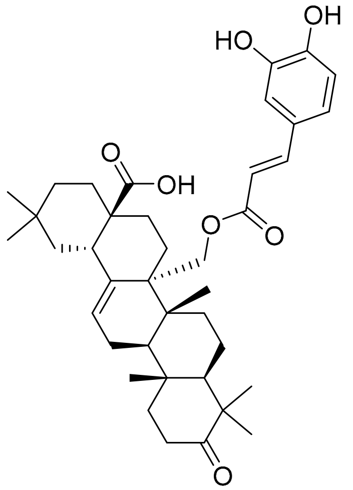
21	Octadecanoic acid	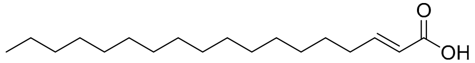
22	Octadecatrienoic acid	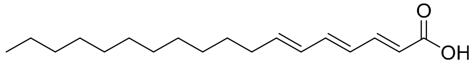
23	Olenoleic acid	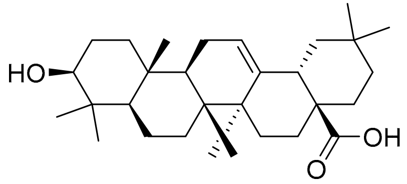
24	Palmitic acid	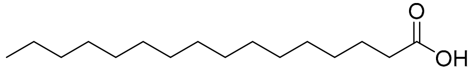
25	Quercetin diglucoside	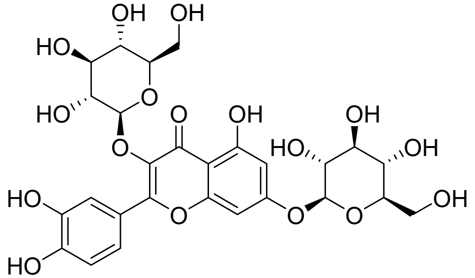

26	Quercetin	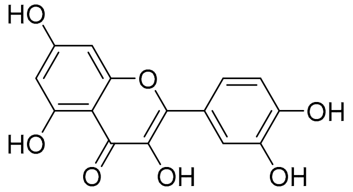
27	Quercetin-3-o-arabinoside	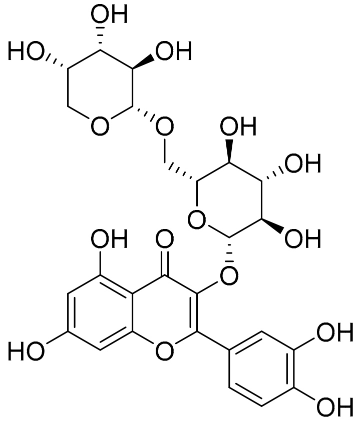
28	Rosmarinic acid	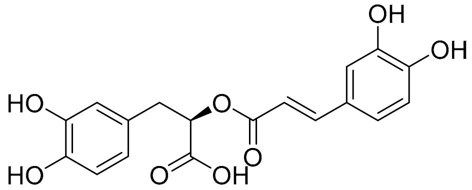

29	Rosmarinic acid-3-glucoside	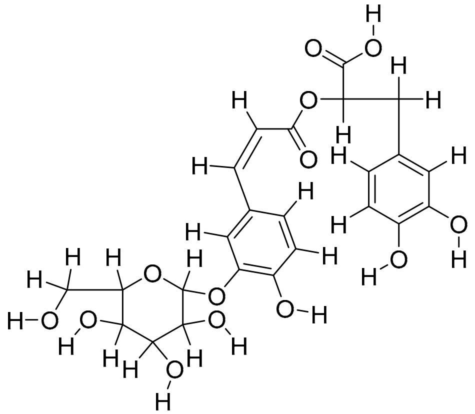
30	Rutin	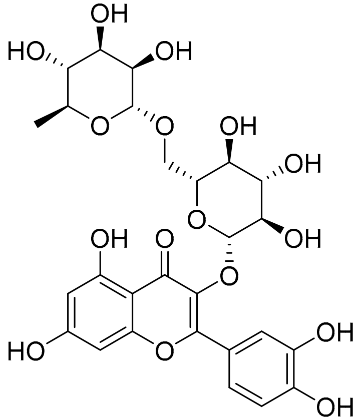
31	Salicylic acid	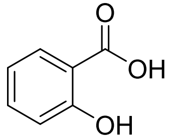

32	Salicylic acid glucoside	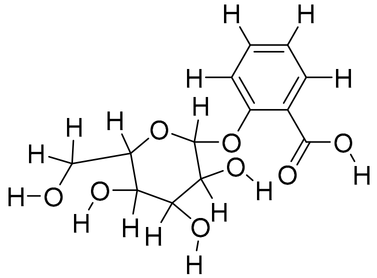
33	Salicylic acid-o-glucoside	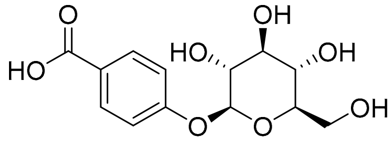
34	Salvialinic acid	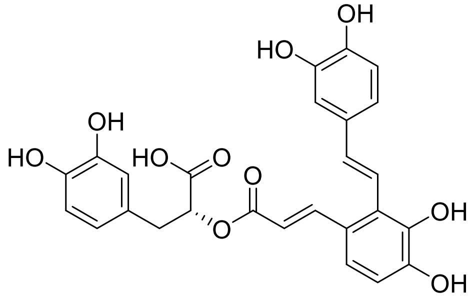
35	Salvianolic acid	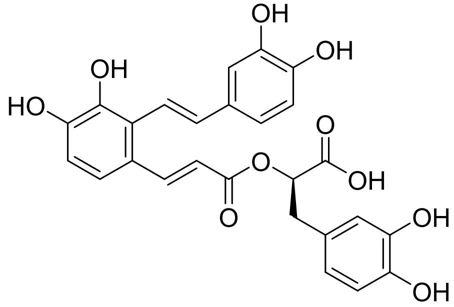

36	Salvianolic acid C	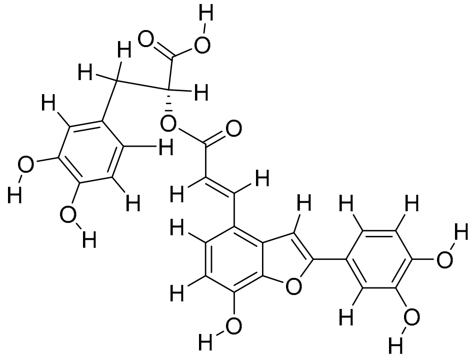
37	Salvianolic acid A	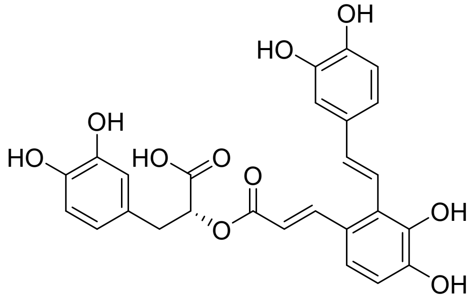
38	Trihydroxy octadecadienoic acid	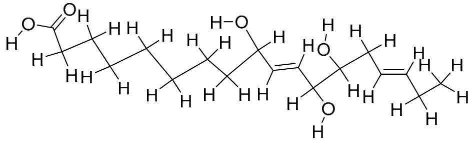
39	Vicenin	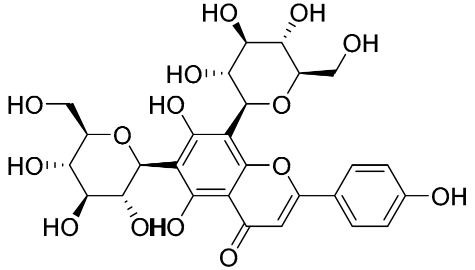
40	Carotene	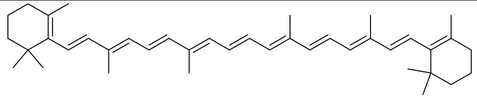

41	Allicin	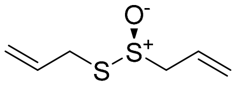
42	Aloin	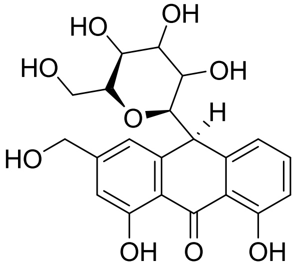
43	Estragole	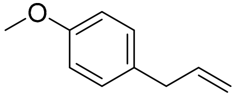
44	Anethole	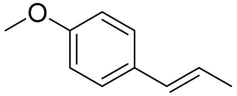
45	Sabinene	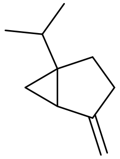
46	Eugenol	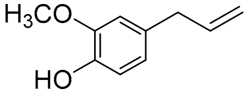
47	Eucalyptol	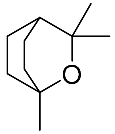
48	Linalool	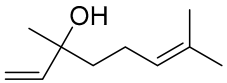
49	Methyl eugenol	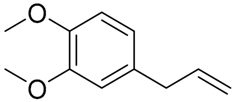

50	Stearic acid	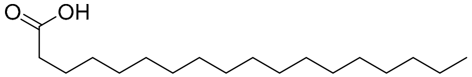
51	Arachidonic acid	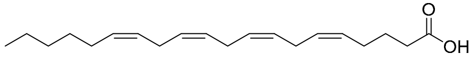
52	Lauric acid	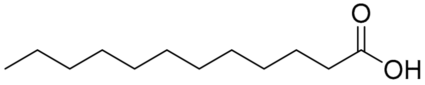
53	Carpic acid	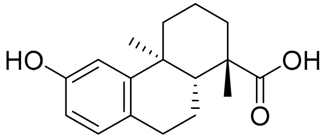
54	α-Linolenic acid	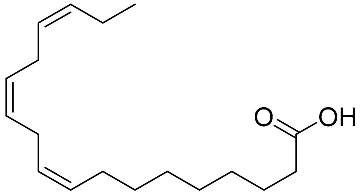
55	Myristicin	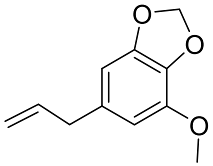
56	Palmitic acid	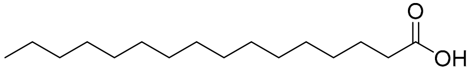
57	Oleic acid	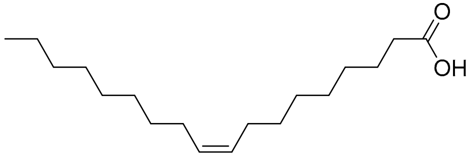
58	Llinalool	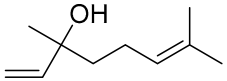

59	Limonene	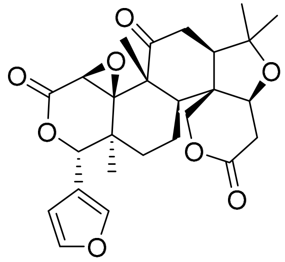
60	Methyl chavicol	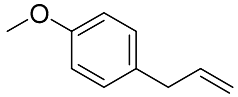
61	β-Pinene	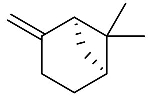
62	α-Pinene	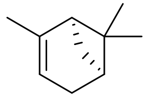
63	Palmatine	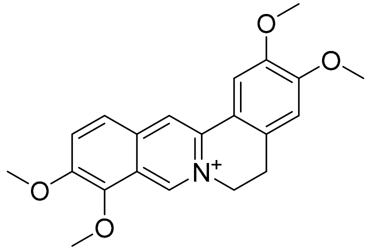
64	Chlorogenic acid	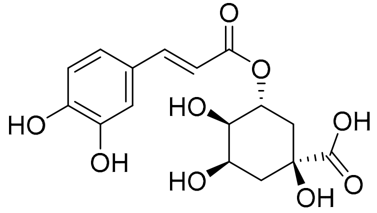

65	Apigenin	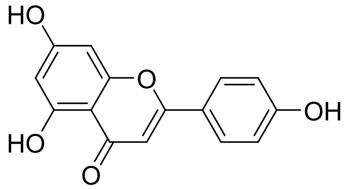
66	Borneol	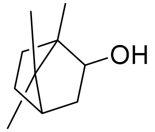
67	o-Cyminate	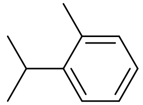
68	Cinnamylacetate	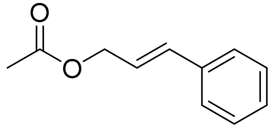
69	Eugenol	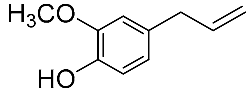
70	Geraneol	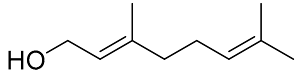
71	B-Caryphyllone	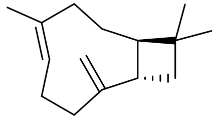
72	β-elemene	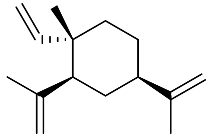
73	Linalool	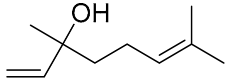

74	Apigenin-7,4-diglucoside	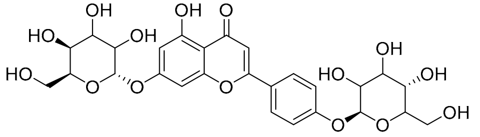
75	methyl cinnamate	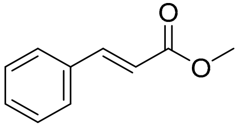
76	1,8-cineole	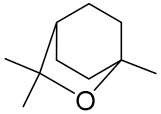
77	Methyl eugenol	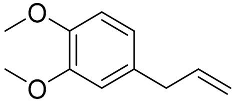
78	beta-sitosterol	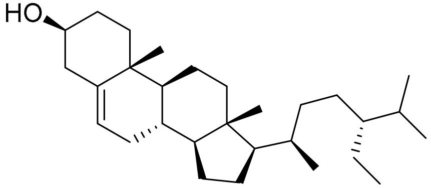
79	Tannin	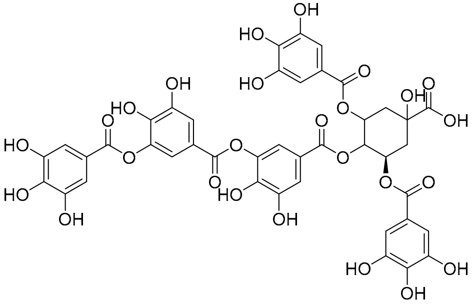

80	ferusoylglucoside	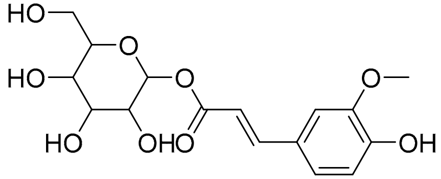
81	Myricetin 3-neohesperidoside	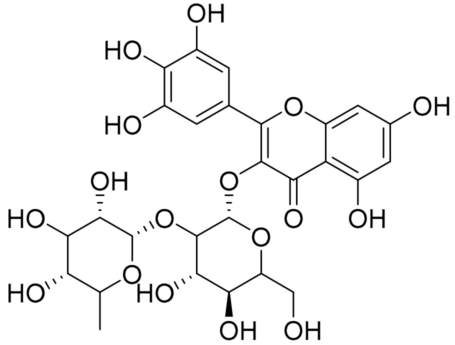
82	Steric acid	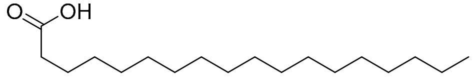
83	Beta-carotene	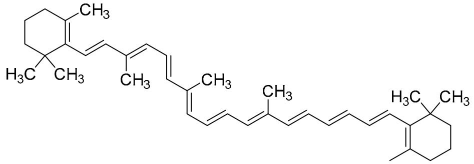
84	Vicine	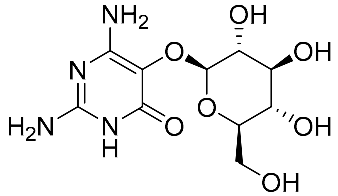
85	Vitamin K	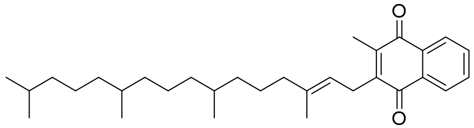

86	Vitamin C	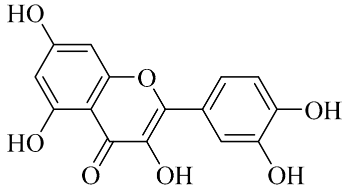
87	Orientin	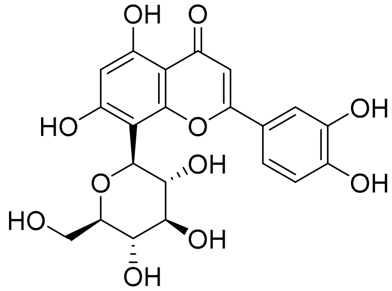
88	Cineole	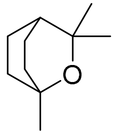
89	Eugenol	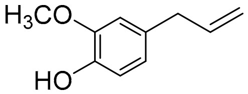
90	Alpha pinene	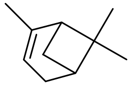
91	Amyrin	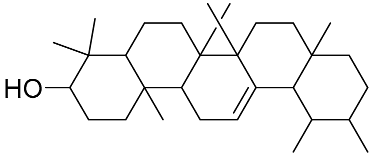
92	Antheole	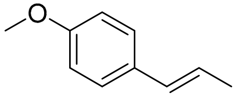

93	Anthocyanin	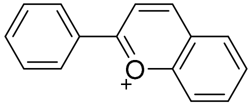
94	Apigenin	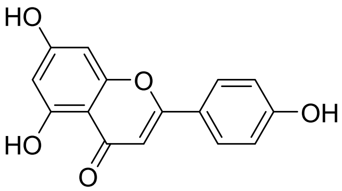
95	Beta Cryptoxanthin	
96	Betulin	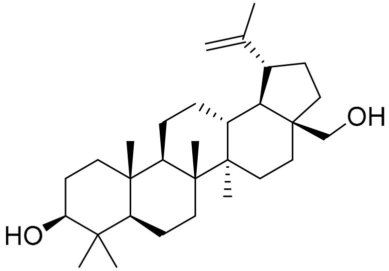
97	Betulinic Acid	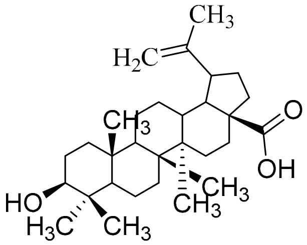

98	Boswellic acid	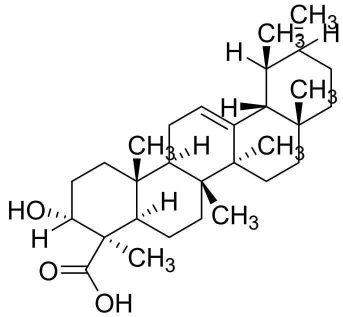
99	Campesterol	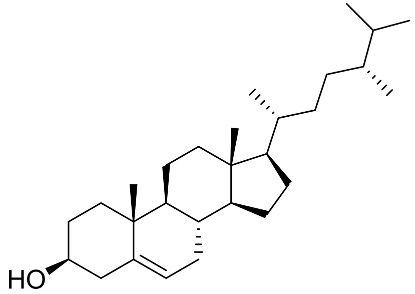
100	Camphene	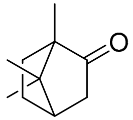
101	Candicine	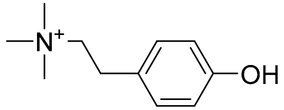
102	car-3-ene	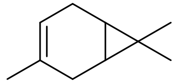

103	Carboxyatractyloside	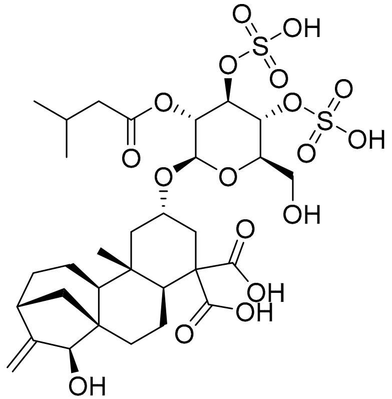
104	Catechol	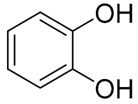
105	Chebulagic Acid	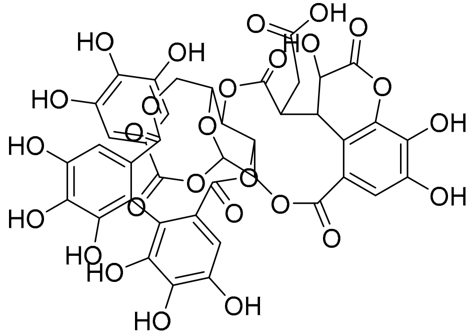
106	Chebulic Acid	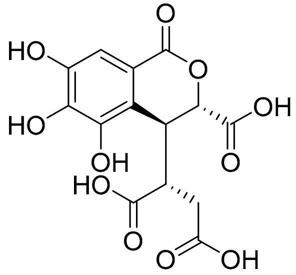

107	Chrysophenol	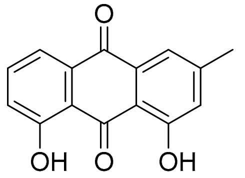
108	Cyanidine-3-rhamnoglucoside	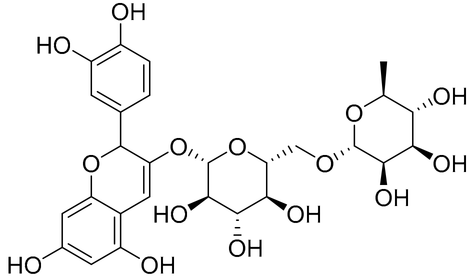
109	Ellagitannin	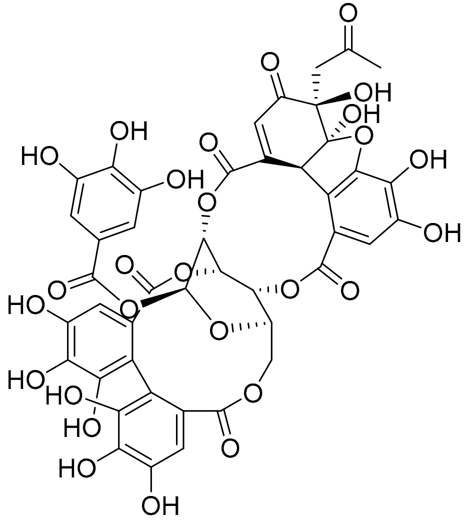
110	Emodin	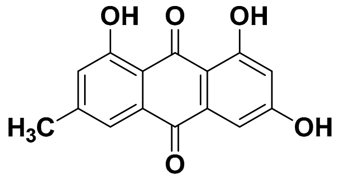

111	Ethyl Gallate	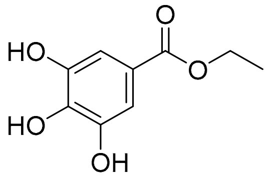
112	Galactomannan	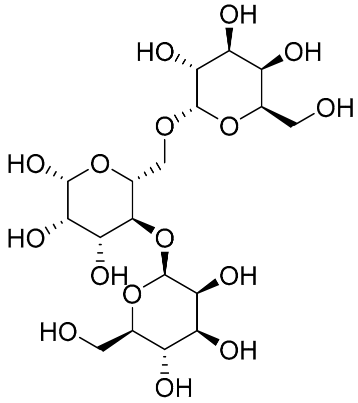
113	Gallotannin	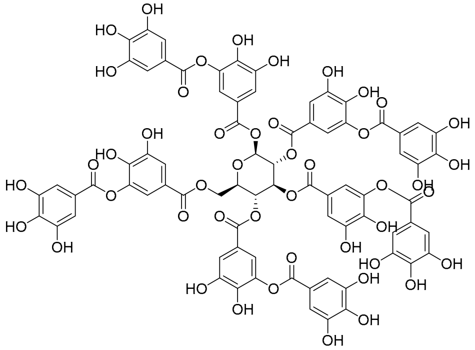

114	Glycyrrhizic Acid	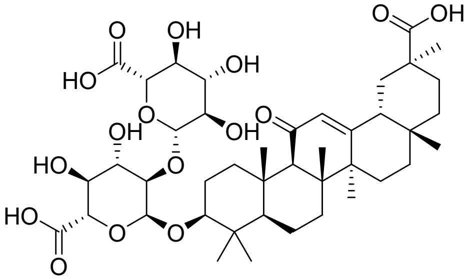
115	Glycyrol	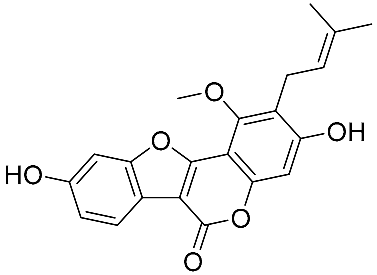
116	Hydroxycinnamic	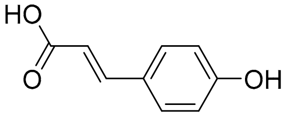
117	Isomahanine	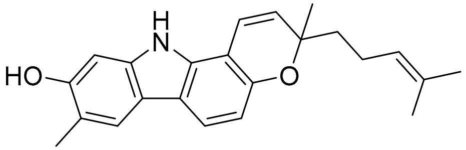
118	Isomethone	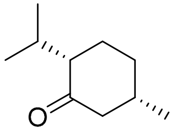

119	Isoorientin	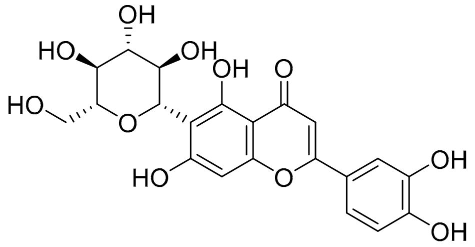
120	Isorhamnatin	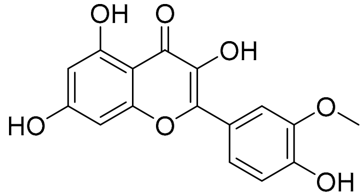
121	limonene	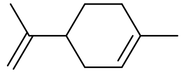
122	linalool acetate	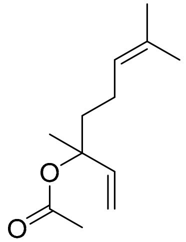
